# A new frontier in therapy personalisation based on in vitro studies to preserve fertility potential of men

**DOI:** 10.1111/and.14244

**Published:** 2021-09-07

**Authors:** Nicola Zampieri, Francesco S. Camoglio, Ilaria Dando

**Affiliations:** ^1^ Woman and Child Hospital Pediatric surgical Unit, Pediatric fertility lab University of Verona Verona Italy; ^2^ Biochemistry Section Department of Neurosciences, Biomedicine and Movement Sciences University of Verona Verona Italy

**Keywords:** hormones, infertility, personalised therapy, testicular cells, therapeutic scheme

## Abstract

At present, there is still a lack of attention to male infertility and fertility impairment. Indeed, the pathologies affecting the reproductive area in man are derived from anatomical or functional alterations of neuroendocrine system; thus, the study of these dysfunctions is necessary for a correct aetiopathogenetic and therapeutic framing of infertile patients. In this article, we underline the importance of the study of the molecular mechanisms regulated by the most common therapy used to treat infertile men, with the aim to highlight the necessity to avoid the administration of the wrong posology or, even more important, the wrong therapy to the patient. Accordingly, we present some pioneer data obtained on primary testicular cells cultured in vitro and treated with human chorionic gonadotropin (hCG). These data pave the way on the possibility to preliminarily test the effectiveness of the therapy in vitro, in order to identify the responsiveness of patient‐derived cells to the treatment and its effectiveness in each subject, in order to identify the correct dosage in a personalised way.

## 1 INTRODUCTION

The development and the functioning of the male genital organs depend on complex neuroendocrine mechanisms, functionally integrated at the level of the GnRH‐gonadotropin‐testicle (GnRH‐Gn‐T) axis, whose integrity is necessary for the normal development of the reproductive and endocrine capacity. The pathologies affecting the reproductive area in men are derived from anatomical or functional alterations of one or more of these structures, the knowledge of which is therefore necessary for a correct aetiopathogenetic and therapeutic framing of these patients (Schlegel et al., 2021).

It is well known that lifestyle is important. In fact, smoking, alcohol abuse, the use of drugs, anabolic substances and a stressful condition can cause a significant decrease in fertility potential of men.

Endocrinologically, gonadotropin‐releasing hormone (GnRH), a decapeptide encoded by a gene located on the short arm of chromosome 8, acts on pituitary gonadotropic cells, stimulating them to secrete luteinising hormone (LH) and follicle‐stimulating hormone (FSH). GnRH secretion is pulsatile, being a fundamental pre‐requisite for a proper reproductive function. FSH and LH, produced respectively by the beta and gamma cells of the adenohypophysis, act on the testes. FSH acts by promoting the early maturative phases of spermatogenesis and by promoting the synthesis of testosterone‐binding protein (ABP), while LH has the role of stimulating the final phases of spermatogenesis and testicular secretion of hormones. Other hormones that exert an action on the andrological axis in male are inhibin B, INSL3 and prolactin (PRL). In the treatment of male infertility, the use of gonadotropins FSH and LH is contemplated and proposed, combined with different therapeutic schemes at different dosages and for a variable period of time in case of altered spermatogenesis or in case of azoospermia. Stimulation should be carried out for at least 3–4 months, until spermatogenesis is achieved. In cases of unsatisfactory responses, the dosage should be rescheduled. Once the restoration of spermatogenesis is obtained, pregnancy is attempted by natural way or, if indicated, by assisted medical procreation techniques.

Alterations in the development and function of the hypothalamic‐pituitary‐testicular axis lead to changes in testicular function and are therefore responsible for hypogonadism, defined as a clinical syndrome that originates from the insufficient ability of the testis to produce physiological levels of testosterone and spermatozoa due to alteration at one or more levels of the axis. This condition may have different causes and manifests itself in different forms depending on the period of onset. From a pathogenetic point of view, hypogonadism can be primary or hypergonadotropic, with alterations in Leydig cells, compromising the production of androgens (testosterone), and/or of seminiferous tubules, resulting in oligospermia or azoospermia. This form is characterised by high gonadotropin levels and low testosterone concentrations. On the contrary, secondary or hypogonadotropic hypogonadism presents alterations in the hypothalamus or pituitary gland that impaired gonadotropin secretion and consequently testicular function. In these patients, reduced levels of gonadotropins and testosterone are observed. Finally, there is hypogonadrenia characterised by altered response to androgens, in which, due to an alteration in the receptor protein or due to enzymatic defects, the response to androgens produced by the testis is reduced or absent. These cases are characterised by normal FSH levels with elevated LH concentrations and normal or elevated testosterone.

Therefore, it is essential to study and understand the biochemistry underlying testicular cell function to better tailor different therapies. Starting from these assumptions, our line of research is focused on the study of testicular cells of a patient, with the aim to investigate regulatory mechanisms and to preserve the fertility potential of men in a personalised way. To deeply study the biological features and hormonal regulation of testicular cells starting from patients' biopsies, it is necessary to perform in vitro analyses. However, although some papers have been published on the isolation of Leydig and Sertoli cells derived from rats and mice (Bhushan et al., 2016; Chang et al., 2011) and fewer on human cells (Gaur et al., 2018), the optimisation of a processing protocol appears to be a priority. In line with this, we have recently developed a procedure that permits to separate and culture in vitro Leydig and Sertoli cells starting directly from a small biopsy of testicular tissue obtained from both paediatric and adult patients.

## 2 MATERIALS AND METHODS

This prospective observational study was approved by the Pediatric Fertility Lab Internal Review Board (IRB CESC 2032).

Since May 2019, at our Fertility Lab Tissue Biobank, we collect scrotal fat, testicular and gubernaculum tissue from paediatric and adult patients operated for testicular pathologies (undescended testes, testicular torsion or azoospermia). Each patient is catalogued with a progressive number and identification. The tissue database is composed of samples from patients aged between 1 and 31 years. Oral and written consent were obtained from each patient or from patients’ parents. At present, we have 54 patients and we analysed a sample of seven different paediatric cases (undescended testes). Normally, after orchidopexy, in those patients with testicular hypotrophy, we perform a treatment with HCG with a standard dosage for 6 weeks. HCG treatment is also used and suggested for infertility in those patients with azoospermia or oligoasthenospermia (Zampieri et al., 2018, 2019).

In order to affirm that the isolated cells are representative of the testicular cell populations, the analysis of mRNA and protein expression levels of their specific markers is fundamental. Indeed, we analysed the expression of luteinising hormone receptor (LHR), which is expressed by Leydig cells, expression of follicle‐stimulating hormone (FSHR), and specific of Sertoli cells.

## 3 RESULTS

**FIGURE 1:**
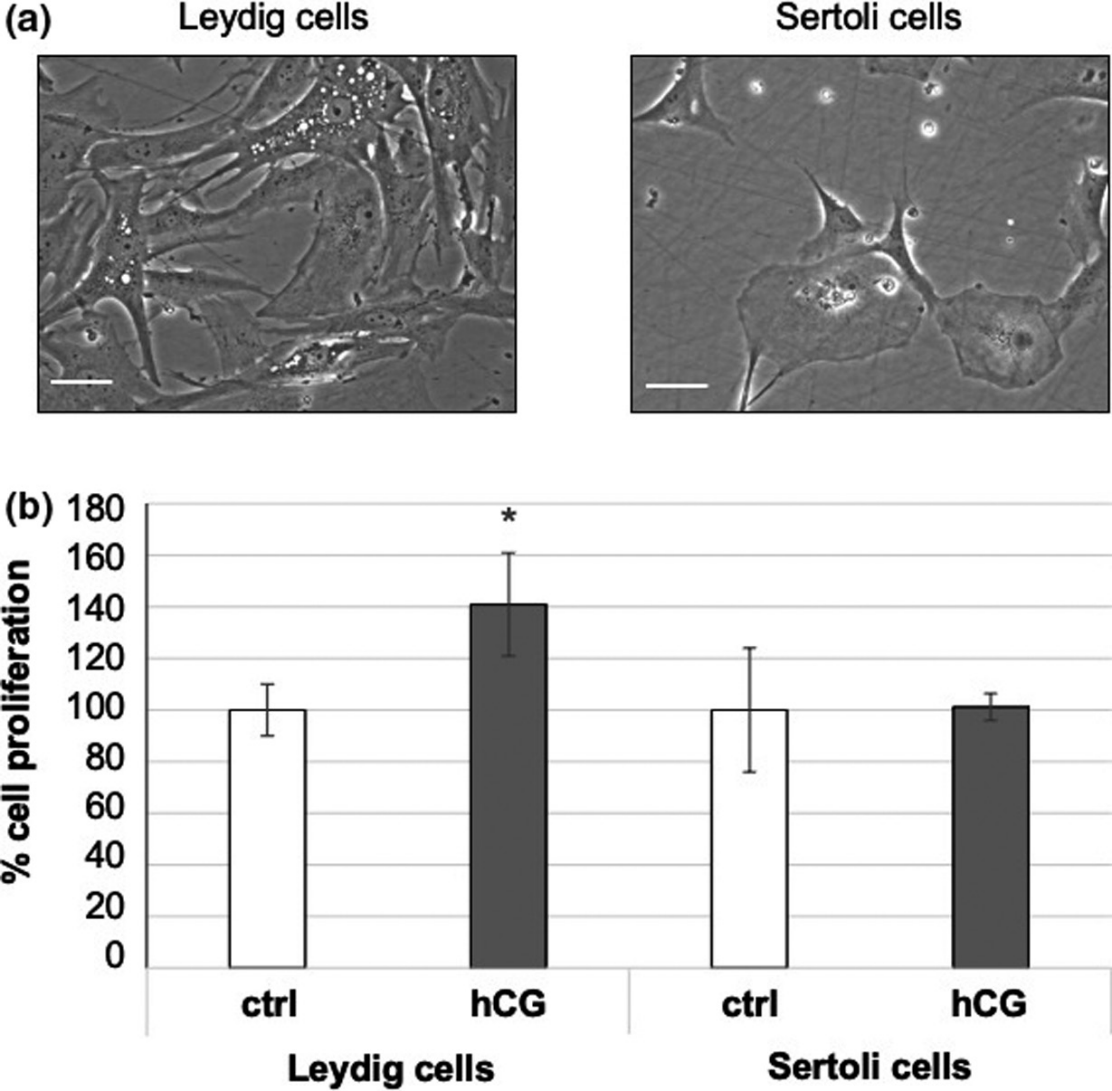
(a) Representative images of Leydig and Sertoli cells derived from a paediatric patient biopsy. Scale bar: 25 µm. (b) Cell proliferation of Leydig and Sertoli cells cultured at 37℃ with 5% CO_2_ in a FBS‐deprived medium (DMEM‐Glutamax + 50 µg/ml gentamicin sulphate) treated with 100 UI/ml hCG once a week for 4 weeks. Cell proliferation was measured by crystal violet assay, and absorbance was measured by spectrophotometric analysis (A595_nm_). Values are the means (±*SD*) of three independent biological replicates. Statistical legend: *p* <.5 (*) of treated cells relative to untreated cells (control)

Among our preliminary results, an important evaluation is the analysis of cell morphology: On the one hand, Leydig cells show a spindle‐like shape with evident white spots inside the cytoplasm, corresponding to lipid droplets, a typical characteristic of active Leydig cells (Figure 1a). On the other hand, Sertoli cells appear to be more round shaped with bigger dimensions (Figure 1a), as it generally happens in vivo. To study the functionality of the isolated cell populations, we treat in vitro the two cell types with human chorionic gonadotropin (hCG), a hormone that specifically binds to LHR, at different doses and at different time points. As reported in Figure 1b, only Leydig cells respond to hCG treatment by showing increased cell proliferation, whereas Sertoli cells are not affected by the treatment. Another important evaluation that we have confirmed also in this cellular system is that, in order to study the real effect of hCG on cell culture, it is necessary to treat the cells in the absence of foetal bovine serum (FBS), which includes LH and FSH (Dando et al., 2021). Among our preliminary series, we found that each cell population responded differently to different dosage schemes, and these differences are visible between different patients' cells, correlating with age (data not yet published). Overall, these data represent an initial, although important, step in the study of the biological mechanisms in Leydig and Sertoli cell dysfunctions that are still obscure, highlighting the enormous potentiality of the in vitro culture of testicular cells.

## 4 CONCLUSION

The lack of attention to male infertility and male impaired fertility potential with respect to female research seems to be the key point to future studies, and the diversity of treatment for and dosages of gonadotropins can create different results generating confusion and uncertainty (Attia et al., 2013; Jung & Seo, 2014).

The basis of our study, and especially our preliminary results, offers important insights for the therapy of the future: Is it possible that each individual patient may benefit from different dosages of gonadotropins compared to patients with similar characteristics? Can we predict the dosages the individual patient need to be administered to obtain a satisfactory result without overdosing? Can we use different dosage schemes based on different pathologies?

The new frontier of personalised therapy could be the testicular biopsy, followed by in vitro treatment of the isolated cells with different dosages to obtain the best proliferation rate. Once the in vitro data are obtained, we can prospect to administer the correct dosage to the individual patient with a targeted dosage therapy. This approach could give several benefits such as avoiding the use of the wrong posology or therapeutic approach and the losing of precious time in the amelioration of testicular volume in kids and in the achievement of pregnancy in adults, together with the relief of the clinical assistance and costs.

## CONFLICT OF INTEREST

The authors declare that they have no conflict of interest to disclose.

## AUTHOR CONTRIBUTIONS

ZN and DI contributed to conceptualisation, data curation, analysis and writing. CFS reviewed the manuscript. All authors have read and approved the final version of the manuscript and agree with the order of presentation of the authors.

## Data Availability

The data that support the findings of this study are available upon request from the corresponding author. The data are not publicly available due to privacy or ethical restrictions.
